# Rapid and Selective Absorption of Plant Defense Compounds From the Gut of a Sequestering Insect

**DOI:** 10.3389/fphys.2022.846732

**Published:** 2022-03-03

**Authors:** Zhi-Ling Yang, Fabian Seitz, Veit Grabe, Sandor Nietzsche, Adrian Richter, Michael Reichelt, Rolf Beutel, Franziska Beran

**Affiliations:** ^1^Research Group Sequestration and Detoxification in Insects, Department of Insect Symbiosis, Max Planck Institute for Chemical Ecology, Jena, Germany; ^2^Department of Evolutionary Neuroethology, Max Planck Institute for Chemical Ecology, Jena, Germany; ^3^Elektronenmikroskopisches Zentrum, Universitätsklinikum Jena, Jena, Germany; ^4^Institut für Zoologie und Evolutionsforschung, Friedrich-Schiller-Universität Jena, Jena, Germany; ^5^Department of Biochemistry, Max Planck Institute for Chemical Ecology, Jena, Germany

**Keywords:** adaptation, cuticle, foregut, glucosinolate, plant defense, sequestration

## Abstract

Many herbivorous insects exploit defense compounds produced by their host plants for protection against predators. Ingested plant defense compounds are absorbed *via* the gut epithelium and stored in the body, a physiological process that is currently not well understood. Here, we investigated the absorption of plant defense compounds from the gut in the horseradish flea beetle, *Phyllotreta armoraciae*, a specialist herbivore known to selectively sequester glucosinolates from its brassicaceous host plants. Feeding experiments using a mixture of glucosinolates and other glucosides not found in the host plants showed a rapid and selective uptake of glucosinolates in adult beetles. In addition, we provide evidence that this uptake mainly takes place in the foregut, whereas the endodermal midgut is the normal region of absorption. Absorption *via* the foregut epithelium is surprising as the apical membrane is covered by a chitinous intima. However, we could show that this cuticular layer differs in its structure and overall thickness between *P. armoraciae* and a non-sequestering leaf beetle. In *P. armoraciae*, we observed a thinner cuticle with a less dense chitinous matrix, which might facilitate glucosinolate absorption. Our results show that a selective and rapid uptake of glucosinolates from the anterior region of the gut contributes to the selective sequestration of glucosinolates in *P. armoraciae.*

## Introduction

Herbivorous insects evolved diverse strategies to cope with defense compounds present in their host plants (Pentzold et al., 2014; Heidel-Fischer and Vogel, 2015; Rashid War et al., 2018). While some insects metabolize and excrete ingested plant defense compounds, others accumulate them in their body and thereby protect themselves against generalist natural enemies (Opitz and Müller, 2009; Heckel, 2014; Petschenka and Agrawal, 2016). The latter strategy, known as sequestration, requires physiological adaptations that enable the transport of ingested defense compounds from the gut to storage sites in the body cavity (Duffey, 1980; Kuhn et al., 2004; Petschenka and Agrawal, 2016). However, the transport mechanisms for plant defense compounds in sequestering insects are not well understood (Erb and Robert, 2016; Petschenka and Agrawal, 2016).

The gut epithelium is the major selective barrier between the environment and the hemocoel, controlling which compounds can enter the body and which cannot (Huang et al., 2015; Denecke et al., 2018). The permeability of the gut epithelium toward plant defense compounds can thus be expected to differ between sequestering and non-sequestering insect species (Duffey, 1980). Such differences were observed for the toxic cardiac glycoside ouabain, which can be absorbed across the gut of the sequestering milkweed bug *Oncopeltus fasciatus*, but not in the case of two non-sequestering insect species (Scudder and Meredith, 1982). Why some insects can absorb cardiac glycosides while others cannot is unknown. The membrane lipid composition has been proposed to play a role as cardiac glycosides are presumably passively absorbed across the gut (Scudder and Meredith, 1982). Another way to prevent the passive absorption of less polar defense compounds, such as ouabain, is their active excretion from gut epithelial cells by efflux carriers (Agrawal et al., 2012).

Transport mechanisms for polar plant defense compounds that cannot passively diffuse across membranes have been investigated in the leaf beetle subfamily Chrysomelinae. Some of the species rely on their food plant to obtain defense compounds, while others synthesize defense compounds *de novo*. Physiological assays demonstrated that the gut epithelium of sequestering and non-sequestering beetle species is similarly permeable for compounds resembling both plant-derived and endogenously produced defense compounds. This showed that the absorption of compounds across the gut epithelium is not highly selective (Discher et al., 2009). After initial absorption or *de novo* synthesis, chrysomeline larvae selectively accumulate defense compounds in exocrine glands (Kuhn et al., 2004; Discher et al., 2009). Molecular studies indicate a selective import of defense compounds into these glands that is presumably mediated by sugar transporters (Strauss et al., 2013; Schmidt et al., 2019).

The horseradish flea beetle, *Phyllotreta armoraciae*, selectively sequesters glucosinolates, the characteristic defense compounds of its brassicaceous host plants (Yang et al., 2020). In plants, glucosinolates form a two-component defense against non-adapted herbivores and pathogens together with plant β-thioglucosidase enzymes (myrosinases; Hopkins et al., 2009; Jeschke et al., 2017; Jeschke and Burow, 2018; Chen et al., 2020; van den Bosch et al., 2020). This defense is activated upon tissue damage bringing glucosinolates into contact with myrosinases, which results in the formation of toxic glucosinolate hydrolysis products (Kissen et al., 2009; Wittstock et al., 2016). Both *P. armoraciae* larvae and adults are able to sequester high amounts of ingested glucosinolates in the hemolymph and additionally possess endogenous myrosinase activity, allowing them to exploit sequestered glucosinolates for their own purposes (Sporer et al., 2020; Yang et al., 2020).

To accumulate ingested glucosinolates in the hemolymph, *P. armoraciae* must be able to prevent hydrolysis by plant myrosinases in the gut. A previous study suggests that *P. armoraciae* can prevent glucosinolate hydrolysis by at least two mechanisms: a rapid absorption of ingested glucosinolates across the gut epithelium and by inactivating plant myrosinases in the gut (Sporer et al., 2021). Rapid absorption of glucosinolates from the gut is likely mediated by membrane transporters. Previously, we identified a family of glucosinolate-specific membrane transporters in *P. armoraciae* (PaGTRs), which belong to the major facilitator superfamily (MFS), more specifically to the sugar porter family (Yang et al., 2021). Most *PaGTR* genes are specifically expressed in the Malpighian tubules, where several of them have been shown to mediate glucosinolate reabsorption. Interestingly, two *PaGTR*s, *PaGTR2* and *PaGTR3*, are expressed in several other tissues including the foregut and hindgut, but not in the midgut. The presence of glucosinolate-specific transporters in the foregut suggests that this tissue plays a role in glucosinolate absorption; however, silencing *PaGTR2* and *PaGTR3* expression using RNA interference had no influence on the uptake of ingested glucosinolates (Yang et al., 2021). It thus remained unknown how and where *P. armoraciae* absorbs ingested glucosinolates from the gut.

The aim of this study is to determine whether the uptake of glucosinolates from the gut is selective and whether glucosinolates are absorbed across the foregut and/or across the midgut epithelium. We obtained evidence that *P. armoraciae* is able to absorb glucosinolates from the foregut, which usually does not play a role in the absorption of hydrophilic compounds. As the foregut is of ectodermal origin and therefore lined with a chitinous intima, we investigated its structure in *P. armoraciae* and also in a closely related sequestering leaf beetle (*Psylliodes chrysocephala*) and a non-sequestering species of the same family (*Phaedon cochleariae*). To identify candidate glucosinolate transporters in the foregut, we compared the expression of genes encoding putative MFS transporters in the foregut, midgut, hindgut, and Malpighian tubules. Our results lay the groundwork for molecular studies of glucosinolate transport across the gut epithelium in *P. armoraciae*.

## Materials and Methods

### Insects and Plants

*Phyllotreta armoraciae* (*P. armoraciae* in the following) was reared on *Brassica juncea* cultivar “Bau Sin” or *Brassica rapa* cultivar “Yu Tsai Sum” (Known-You Seed Co., Ltd., Kaohsiung) plants in mesh cages in a controlled-environment chamber (24°C, 60% relative humidity, 14 h light/10 h dark period). Food plants were cultivated in a controlled-environment chamber (24°C, 55% relative humidity, 14 h light/10 h dark period). Adults were supplied with 3-week-old potted plants for feeding and egg laying. After 1 week, adults were supplied with new plants and plants with eggs were placed in a separate cage for larval development, which takes 2 to 3 weeks under these conditions. Three weeks later, the remaining plant material was discarded and the soil containing the pupae was kept in plastic containers (9 l volume, Lock&Lock, Seoul, South Korea). These containers were checked every 2–3 days for newly emerged beetles and the soil was moistened with water to prevent desiccation.

*Psylliodes chrysocephala* (*Ps. chrysocephala* in the following) was reared on *B. rapa* plants in mesh cages in a controlled-environment chamber (23°C, 75% relative humidity, 14 h light/10 h dark period). The rearing protocol is similar to that of *P. armoraciae.* As the larval development of *Ps. chrysocephala* takes longer compared to that of *P. armoraciae*, we removed the above-ground plant material after 4 weeks instead of 3 weeks.

*Phaedon cochleariae* (*Ph. cochleariae* in the following) was reared on Chinese cabbage, *Brassica rapa* ssp. *pekinensis*, bought in a local supermarket. All life stages were kept together in plastic containers in a controlled-environment chamber (15°C, 60% humidity, 16 h light/8 h dark period).

The myrosinase-deficient *Arabidopsis thaliana tgg1 × tgg2* double knockout mutant was cultivated in a controlled-environment chamber under short day conditions (21°C, 55% relative humidity, 10 h light/14 h dark period).

### Chemicals

2-Propenyl (2Prop) glucosinolate was purchased from Roth (Mannheim, Germany), 4-methylsulfinylbutyl (4MSOB) glucosinolate was purchased from Phytoplan (Heidelberg, Germany), and 4-hydroxybenzyl (4OHBenz) glucosinolate was isolated from *Sinapis alba* seeds following Thies (1988). The phenolic glucoside salicin and the iridoid glucoside catalpol were purchased from Sigma-Aldrich (Steinheim, Germany); the cyanogenic glucoside linamarin was purchased from Biozol (Eching, Germany).

### Selectivity of Glucoside Uptake

To determine whether ingested glucosides are selectively absorbed across the gut, we fed three-day-old adult *P. armoraciae* beetles (reared on *B. rapa*) with 101.2 nl of an aqueous mixture of three glucosinolates and three non-host glucosides (see section Chemicals; each at 1.5 mM) using a Nanoliter 2010 Injector (World Precision Instruments, Sarasota, FL, United States). To visualize the uptake into the gut and confirm that the gut tissue was not damaged during dissection, we added 0.2% (w/v) amaranth dye to the mixture. 4 min after ingestion, we dissected the beetle into head, gut, and the remaining body. The dissected gut was washed twice in *ca.* 15 ml of phosphate-buffered saline (PBS; pH 6.8) before sampling. As controls, we dissected beetles that had not been fed (*n* = 10 per treatment, tissues of three beetles per replicate). Samples were immediately homogenized in 80% (v/v) methanol using a plastic pestle, frozen in liquid nitrogen, and stored at −20°C until extraction. Homogenized samples were vortexed for 30 s and centrifuged (10 min, 16,000 × *g*, 4°C). The supernatant was transferred to a new reaction tube, dried using vacuum centrifugation, and re-dissolved in 60 μl ultrapure water. Samples were stored at −20°C until they were analyzed using liquid chromatography coupled with tandem mass spectrometry (LC–MS/MS). To determine the recovery rate of fed glucosides from beetle tissue extracts, we additionally extracted aliquots of the glucoside mixture fed to the beetles as described above. The recovery rate was calculated by expressing the total detected amount of each glucoside in all extracted beetle tissues (gut, head, and remaining body) relative to the total detected amount of the same compound in control extract, which was set to 100%.

### Comparison of the Glucosinolate Uptake Rate Across the Foregut and the Midgut

To investigate the glucosinolate uptake rate across the foregut, we first determined a time point at which the entire ingested plant tissue is localized in the foregut. To this end, we stopped beetle feeding at different time points, dissected the gut, and determined the localization of the ingested plant tissue under a stereo microscope. With this approach, we found ingested plant material to be localized exclusively in the foregut when beetles were dissected after feeding for 15 s. To determine the glucosinolate uptake rate, we allowed newly emerged *P. armoraciae* beetles (reared on *B. juncea*) to feed for 15 s on a detached *A. thaliana tgg1 × tgg2* rosette leaf from a 6-week-old plant and then immediately separated the head capsule with the attached gut from the remaining body. The gut attached to the head was washed twice in *ca.* 15 ml PBS buffer (pH 6.8). Afterward, the head capsule was discarded and the gut was transferred into a reaction tube. For each sample, tissues of three beetles were pooled (*n* = 10). To determine the background of 4MSOB glucosinolate in beetles, we collected unfed beetles as control (*n* = 10). Samples were frozen in liquid nitrogen and stored at −20°C until extraction.

To determine the glucosinolate uptake rate across the midgut, we injected 101.2 nl of an *A. thaliana tgg1 × tgg2* leaf extract directly into the anterior midgut of living beetles. The leaf extract was prepared by homogenizing a detached rosette leaf from a 6-week old *A. thaliana tgg1 × tgg2* mutant plant using metal beads (5 mm diameter, QIAGEN, Hilden, Germany) for 4 min at 30 Hz in a tissue lyzer (QIAGEN). After centrifugation (10 min, 16,000 × *g*, 4°C), we collected the supernatant and added 0.25% (w/v) amaranth dye to visualize the injection. To inject, we gently pulled the head with forceps to expose the anterior end of the midgut and injected the leaf extract using a Nanoliter 2010 Injector (World Precision Instruments). After 15 s, we dissected the beetle as described above. For each sample, tissues of three beetles were pooled (*n* = 10). Because we detected only traces of 4MSOB glucosinolate in unfed beetles, we did not include an additional negative control in this experiment. All samples were extracted as described above and stored at −20°C until analysis using LC–MS/MS. To determine the recovery rate of injected glucosinolates, we quantified the glucosinolate amount in an aliquot of the injected leaf extract using high performance liquid chromatography coupled with diode array detection (HPLC-DAD) as described in Yang et al. (2021). The recovery rate was calculated by quantifying the total detected amount of 4MSOB glucosinolate in gut and the remaining body extracts relative to the glucosinolate amount in the injected leaf extract, which was set to 100%. On average, we recovered between 26 and 55% of the injected 4MSOB glucosinolate from the tissue extracts.

### LC–MS/MS Analysis

The levels of glucosinolates and non-host glucosides in tissue extracts were quantified using an Agilent 1200 HPLC system (Agilent, Santa Clara, CA, United States) coupled to an API5000 or an API3200 tandem mass spectrometer (AB SCIEX, Darmstadt, Germany). Glucosinolates were separated on an EC 250/4.6 NUCLEODUR Sphinx RP column (250 mm × 4.6 mm, 5 μm; Macherey-Nagel, Düren, Germany). The mobile phase consisted of 0.2% (v/v) formic acid in ultrapure water as solvent A and acetonitrile as solvent B, at a flow rate of 1 ml/min. The elution gradient was as follows: 0–1 min, 1.5% B; 1–6 min, 1.5–5% B; 6–8 min, 5–7% B; 8–18 min, 7–21% B; 18–23 min, 21–29% B; 23–23.1 min, 29–100% B; 23.1–24 min, 100% B; 24–24.1 min, 100–1.5% B; and 24.1–28 min, 1.5% B. The ionization source was set to negative mode. The ion spray voltage was maintained at −4,500 eV. Gas temperature was set to 700°C, nebulizing gas to 70 psi, drying gas to 60 psi, curtain gas to 20 psi, and collision gas to 10 psi. Non-host glucosides were separated on an Agilent XDB-C18 column (5 cm × 4.6 mm, 1.8 μm, Agilent, Waldbronn, Germany). The mobile phase consisted of 0.05% (v/v) formic acid in ultrapure water as solvent A and acetonitrile as solvent B, at a flow rate of 1.1 ml/min. The elution gradient was as follows: 0–0.5 min, 5% B; 0.5–2.5 min, 5–31% B; 2.5–2.52 min, 31–100% B; 2.25–3.5 min, 100% B; and 3.5–3.51 min, 100–5% B, 3.51–6 min, 5% B. The ion spray voltage was maintained at −4,200 eV. The turbo gas temperature was set to 630°C, nebulizing gas to 60 psi, curtain gas to 30 psi, and collision gas to 5 psi. For each compound, the transitions from precursor ion to product ion were monitored using multiple reaction monitoring (MRM; Supplementary Table 1). Compounds were quantified using external calibration curves. Analyst Software 1.6 Build 3773 (AB SCIEX) was used to acquire and process data.

### Comparison of the Foregut Structure of Sequestering and Non-sequestering Leaf Beetle Species

To further investigate absorption *via* the foregut, we compared its morphological properties in *Ps. armoraciae* with those in another sequestering flea beetle (*Ps. chrysocephala*) and in the non-sequestering chrysomeline species *Ph. cochleariae*. To document the properties of the foregut, we used micro computed tomography (μCT), confocal laser scanning microscopy (CLSM), and transmission electron microscopy (TEM).

For μCT analysis, specimens (with legs removed) were fixed in Duboscq-Brasil fixative for 1 d, washed using 70% (v/v) ethanol, dehydrated with ethanol (70–100%), stained in 1% (w/v) iodine in ethanol for 4 d, and critical-point dried (EmiTech K850 Critical Point Dryer, Quorum Technologies Ltd., Ashford, England). Dried specimens were scanned using a Bruker Skyscan 2211 μCT scanner (Kontich, Belgium), equipped with a high-resolution X-ray sensitive CCD camera. Setting parameters are provided in Supplementary Table 2. Segmentation was done with Amira 6.0.1 software (Thermo Fisher Scientific, Hillsboro, United States).

For CLSM analysis, we placed dissected foregut tissue in glycerol. Fluorescent images were acquired using a Zeiss cLSM 880 (Oberkochen, Germany) equipped with a 10x/0.3 air objective (EC Plan-Neofluar, Zeiss) for overview images and a 40x/1.2 water objective (C-Apochromat, Zeiss) for detailed views. A 405 nm laser diode was used for excitation. Autofluorescence emission was detected between 410 nm and 695 nm. Overviews were acquired as tiled z-stacks with 10% overlap using the ZEN software (black 2.1, Zeiss).

For TEM analysis, we fixed specimens (with legs removed) in Karnovsky’s fixative (4% formaldehyde and 2.5% glutaraldehyde in 0.1 M Na-cacodylate buffer; pH 7.4) for 24 h at room temperature. Afterward, specimens were washed in 0.1 M Na-cacodylate buffer and the head with the foregut was dissected in the same buffer. Post-fixation of the dissected tissue was performed using osmium tetroxide (1% OsO_4_ in 0.1 M Na-cacodylate buffer) for 2 h. Afterward, samples were washed in 0.1 M Na-cacodylate buffer, dehydrated in ethanol (30% for 10 min and 50% for 5 min), stained using 2% uranyl acetate (in 50% ethanol) for 1 h in the dark, dehydrated in ethanol (50–100%), and finally in propylene oxide for 2 min. Samples were transferred into a series of mixtures of Araldite (Plano GmbH, Wetzlar, Germany) and propylene oxide (1:2; 1:1; and 2:1, at least 1 h for each step), and embedded in pure Araldite. The embedded samples were cut using Leica Ultracut S (Leica, Wetzlar, Germany), and scanned using a Zeiss EM 900 TEM with a resolution of 0.6 nm, operated at 80 kV. Photo documentation was done with a TRS slow scan CCD camera system (Moorenweis, Germany).

### Tissue-Specific Expression of Putative MFS Transporter Genes

We previously annotated 222 putative MFS transporters in a gut- and Malpighian tubule-specific transcriptome of adult *P. armoraciae* beetles, and performed RNA-sequencing to investigate their expression in the foregut, midgut, hindgut, and Malpighian tubules, respectively, each with four replicates (Yang et al., 2021). To compare the expression of putative MFS transporter genes across these four tissues, we analyzed the dataset as follows. First, we excluded lowly expressed genes with mean TPM (transcripts per kilobase million) values below 1 in all four tissues (Supplementary Data 1). Genes with a mean TPM value below 1 in one tissue were considered as not expressed in the respective tissue. After sorting MFS transporter genes according to their TPM values within each tissue, we visualized gene expression in the foregut, midgut, hindgut, and Malpighian tubules using a heatmap generated in HemI (Deng et al., 2014).

### Statistical Analysis

Statistical analyses were conducted in SigmaPlot 14.0 (Systat Software Inc., Erkrath, Germany) or in R 4.0.4 (R Core Team, 2021). The recovery of different glucosides fed to the beetles, and the sequestration of different glucosides relative to recovered amounts were compared using one-way ANOVA, followed by post-hoc multiple comparisons test. The sequestration of different glucosides relative to ingested glucosides were compared using generalized least squares method (Pinheiro et al., 2019). The overall gene expression levels of the expressed MFS genes in foregut and midgut were compared using the 141 TPM values from each tissue by Mann–Whitney *U*-test. If necessary, data were transformed prior to analysis. For analyses using the generalized least squares method, varIdent variance structure was used to allow each group to have a different variance. Likelihood ratio tests were applied to obtain *p* values by comparing models with and without explanatory variables (Zuur et al., 2009). Significant differences between groups were revealed with factor level reductions (Crawley, 2013). Glucosinolate uptake rate across the foregut and midgut were compared by Mann–Whitney *U*-test. Information about data transformation, statistical methods, and results of the statistical analyses are summarized in Supplementary Table 3.

## Results

### *Phyllotreta armoraciae* Selectively Absorbs Ingested Glucosinolates Across the Gut

**Figure 1:**
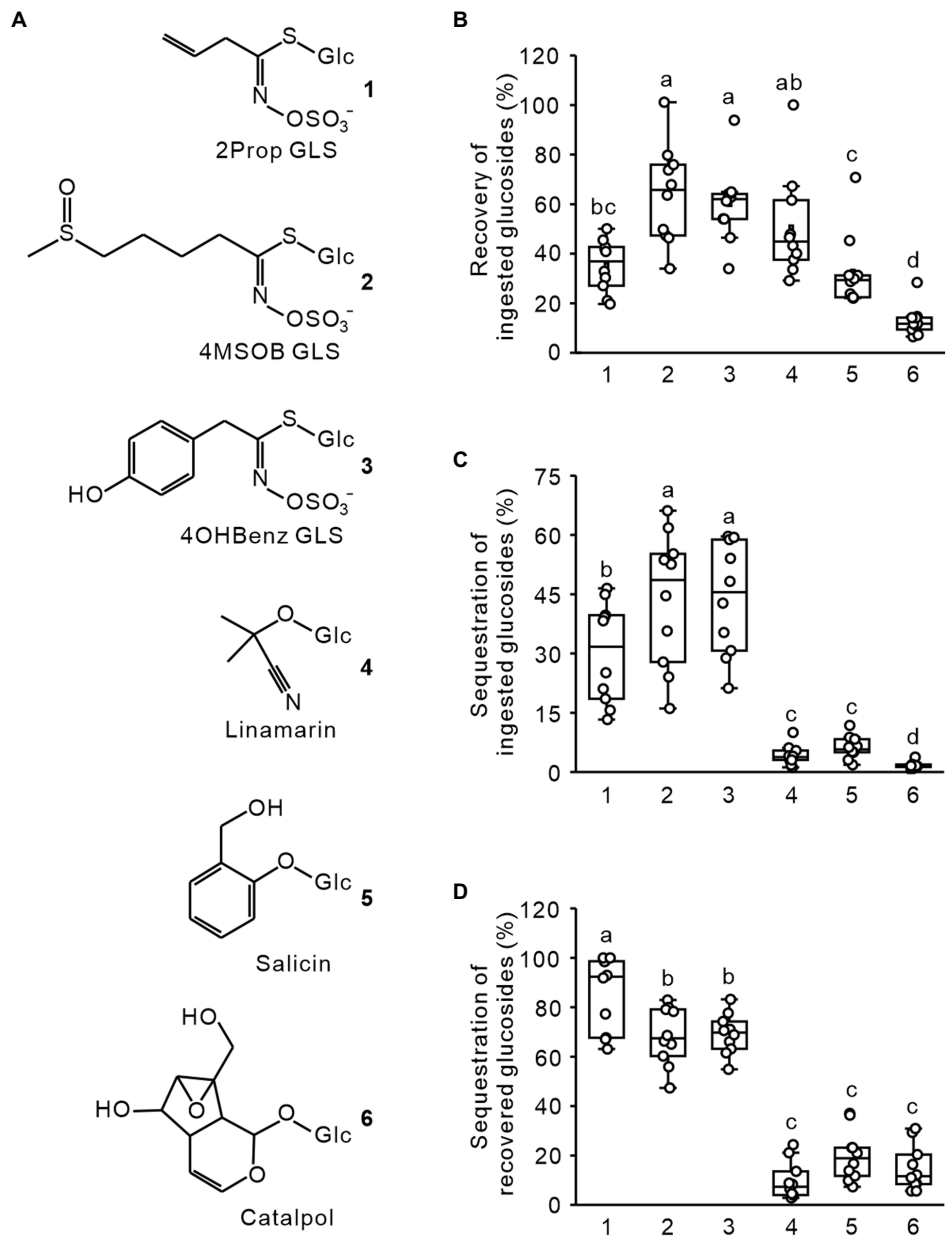
Recovery and sequestration of ingested glucosides in adult *Phyllotreta armoraciae*. **(A)** Chemical structures of glucosinolates (GLS) and non-host glucosides used in feeding assays. **(B)** Recovery of ingested glucosides in *P. armoraciae*. Adult beetles were fed with 101.2 nl of an equimolar mixture of six glucosides. After 4 min, beetles were dissected into head, gut, and the remaining body. Tissues were extracted in 80% (v/v) methanol and the amounts of each glucoside were quantified using LC–MS/MS. To determine the recovery rate, we extracted 101.2 nl of the fed glucoside mixture and quantified the glucoside amounts with the same method. The total amounts of glucosides recovered in tissue extracts (head, gut, and remaining body combined) are expressed relative to the recovery control (set to 100%; *n* = 10). **(C)** The glucoside amount detected in the remaining body was expressed relative to the total ingested glucoside amount (set to 100%; *n* = 10). **(D)** The glucoside amount detected in the remaining body was expressed relative to the total amount detected in head, gut, and remaining body extracts (set to 100%; *n* = 10). Box plots show the median, interquartile range, and outliers of each dataset. Comparisons were conducted using generalized least squares method or one-way ANOVA. Boxplots labeled with different letters are significantly different (*p* < 0.05). 2Prop, 2-propenyl; 4MSOB, 4-methylsulfinylbutyl; and 4OHBenz, 4-hydroxybenzyl.

To investigate whether *P. armoraciae* adults possess a selective uptake mechanism for glucosinolates, we fed beetles with an aqueous equimolar mixture of glucosinolates and non-host glucosides (Figure 1A), dissected them into head, gut, and the remaining body, and quantified the amounts of each glucoside in tissue extracts using LC–MS/MS. The total recovery rate differed significantly among the glucosides (ANOVA, *F* = 22.339, *p* < 0.001; Figure 1B), ranging from only 13% for catalpol to up to 64% for 4MSOB glucosinolate. We then compared how much of each glucoside was sequestered in the beetle body (without head and gut) and found that significantly more ingested glucosinolates had been sequestered than non-host glucosides (Figure 1C; generalized least squares, *F* = 96.645, *p* < 0.001). In addition, the relative distribution of the six glucosides in beetle tissues differed (body versus gut and head; ANOVA, *F* = 97.140, *p* < 0.001), with most glucosinolates found in the body (between 68 and 86%), and most non-host glucosides found in the head and gut (Figure 1D).

### Glucosinolates Are Rapidly Absorbed Across the Foregut

**Figure 2:**
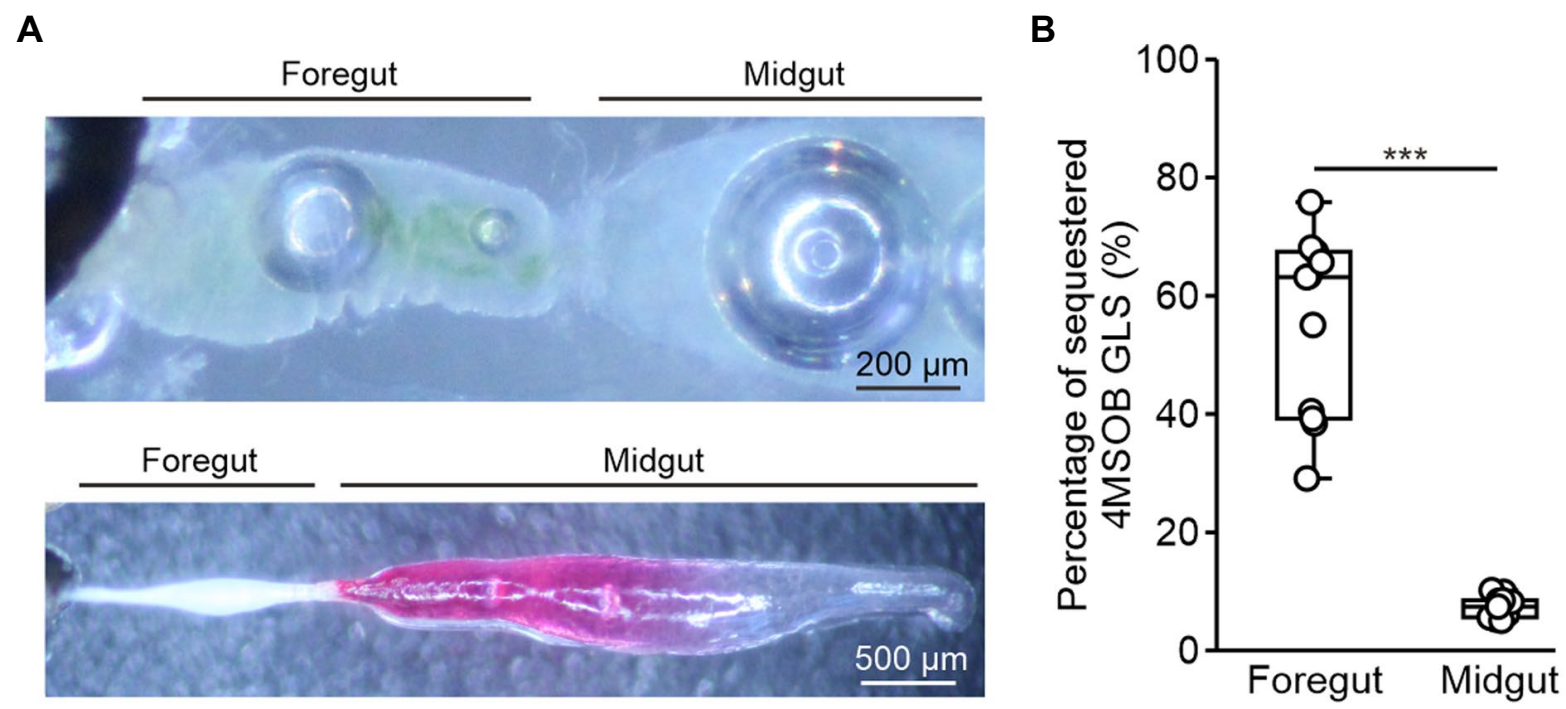
Localization of glucosinolate uptake in adult *Phyllotreta armoraciae*. **(A)** Upper picture: Dissected gut with ingested plant material localized in the foregut. Lower picture: Dissected gut with injected leaf extract visualized using amaranth dye. **(B)** Glucosinolate uptake from the foregut and midgut in *P. armoraciae*. Guts of newly emerged adults were separated from the remaining body after 15 s of feeding or injection. Dissected tissues were extracted using 80% (v/v) methanol and glucosinolates were quantified by LC–MS/MS. The percentages of sequestered glucosinolates were calculated as the glucosinolate amounts in the remaining bodies relative to the total amounts detected in guts and the remaining bodies (Mann–Whitney *U*-test, ^***^ *p* < 0.001, *n* = 10). Box plots show the median and interquartile range of each dataset.

To determine whether ingested glucosinolates are absorbed across the foregut epithelium, we separated the gut and the remaining body of beetles that had fed for 15 s on a leaf of the myrosinase-deficient *A. thaliana tgg1* × *tgg2* mutant. Afterward, we analyzed the distribution of ingested 4MSOB glucosinolate in tissue extracts using LC–MS/MS. At this time point, the ingested plant material was still localized in the foregut (Figure 2A), and 55% of the total detected glucosinolates were found in body extracts (Figure 2B).

To determine the glucosinolate uptake rate from the midgut, we injected an *A. thaliana tgg1* × *tgg2* mutant leaf extract directly into the anterior midgut (Figure 2A) and analyzed the distribution of 4MSOB glucosinolate in the gut and the remaining body 15 s later. In this experiment, only 7% of the total detected 4MSOB glucosinolate were sequestered in the body (Figure 2B). Thus, the glucosinolate uptake rate from the midgut was significantly slower than that from the foregut under our experimental conditions (Mann–Whitney *U*-test, *U* = 0, *p* < 0.001).

### Expression of MFS Transporter Genes in the Foregut

**Figure 3:**
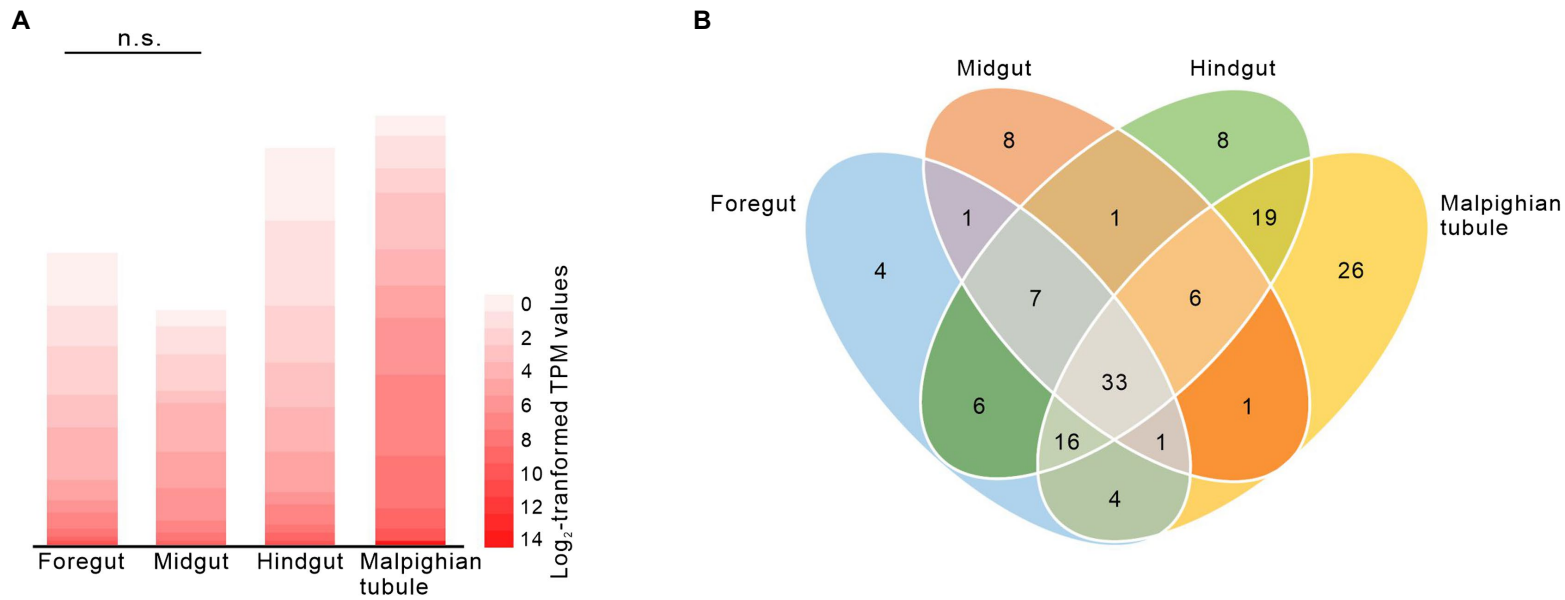
Expression of putative major facilitator superfamily (MFS) genes in the foregut, midgut, hindgut, and Malpighian tubules of adult *Phyllotreta armoraciae* shown by heatmap **(A)** and Venn diagram **(B)**. Expression of 141 putative MFS genes [TPM (transcripts per million) > 1] was analyzed by RNA-sequencing (Yang et al., 2021). Each value represents the mean of four biological replicates (Supplementary Data 1). The overall expression levels of putative MFS genes in foregut and midgut were compared by Mann–Whitney *U*-test. n.s., not significantly different.

To identify candidate transporters involved in glucosinolate absorption across the foregut, we analyzed the expression of putative MFS transporter genes that were previously identified in a gut- and Malpighian tubule-specific transcriptome in the foregut, midgut, hindgut, and Malpighian tubules of adult *P. armoraciae* beetles. From a total of 141 expressed putative MFS transporter genes, 72 were expressed in the foregut, of which nine were expressed at least 2-fold higher in the foregut than in any other tissue (Figure 3; Supplementary Data 1). These nine genes comprised four members of the sugar porter family (2.A.1.1), including the previously identified glucosinolate-specific transporter *PaGTR3*, and the transporter gene with the highest expression level in the foregut, a member of the endosomal spinster family (2.A.1.49) and members of several other MFS transporter families (Supplementary Data 1). The overall expression level of MFS transporter genes in the foregut did not differ from that in the midgut (Figure 3A, Mann–Whitney *U*-test, *U* = 8922, *p* = 0.136).

### The Foregut Structure of Sequestering and Non-sequestering Leaf Beetle Species Differs

**Figure 4:**
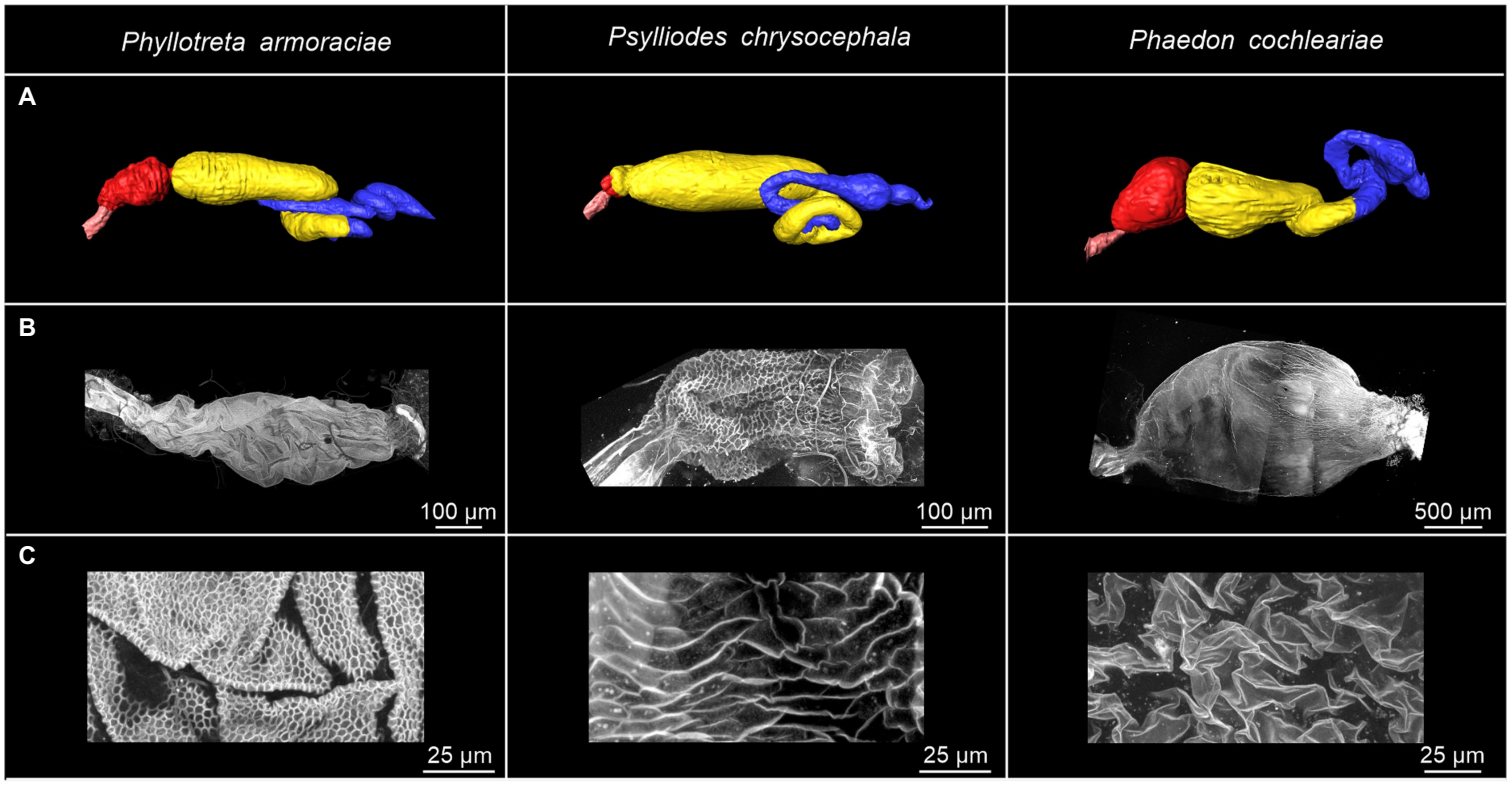
Overview of the gut and foregut structures of *Phyllotreta armoraciae*, *Psylliodes chrysocephala,* and *Phaedon cochleariae* adults. **(A)** 3D reconstruction of the digestive systems. The esophagus, crop, midgut, and hindgut are indicated by pink, red, yellow, and blue coloration, respectively. **(B,C)** Autofluorescence of chitin in isolated foreguts. The excitation of cuticular autofluorescence was conducted by a 405 nm laser. Emission was detected between 410 and 695 nm.

**Figure 5:**
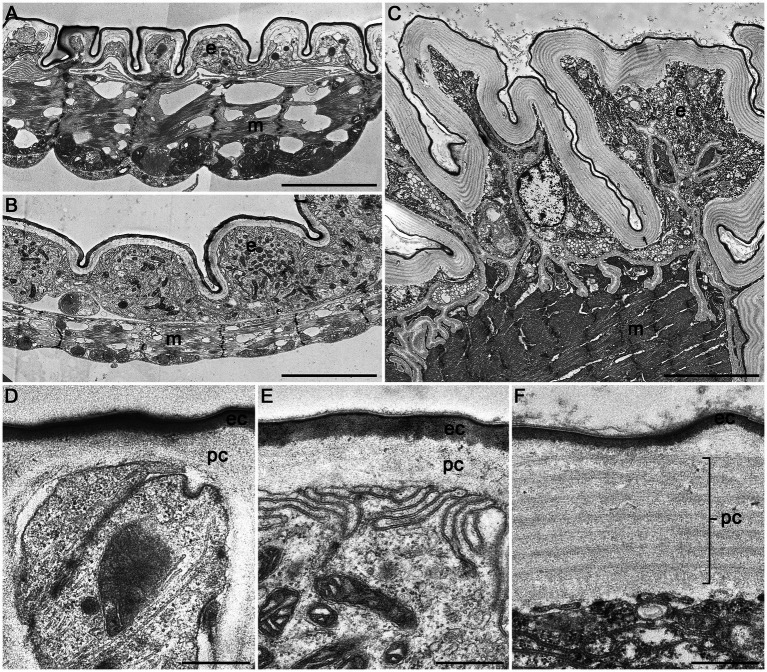
Transmission electron microscopic images of cross sections of the crop of *Phyllotreta armoraciae* **(A,D)**, *Psylliodes chrysocephala* **(B,E)**, and *Phaedon cochleariae* **(C,F)**. A notable difference in overall size is shown in **(A–C)** (same magnification). A continuous epithelial cell layer (e), muscle layer (m), procuticle (pc), and epicuticle (ec) are visible in all three species. Scale bars are 5 μm **(A–C)** and 500 nm **(D–F)**.

3D reconstructions of the entire digestive system revealed distinct differences in the relative size of the foregut between *P. armoraciae*, the closely related glucosinolate-sequestering *Ps. chrysocephala*, and the non-sequestering *Ph. cochleariae*. In particular, the foregut of *Ps. chrysocephala* was very small compared to the foreguts of *P. armoraciae* and *Ph. cochleariae* (Figure 4A). We then visualized the chitinous intima covering the foregut epithelium using CLSM. Chitin autofluorescence appeared “mesh-like” in *P. armoraciae*, “brick-like” in *Ps. chrysocephala*, and as a more continuous layer in *Ph. cochleariae* (Figures 4B,C). Chitin is usually organized in microfibrils that are arranged in horizontal sheets (laminae) in the procuticle. In TEM analyses, chitin laminae were clearly visible in the procuticle of *Ph. cochleariae*, but not in the much thinner procuticles of *P. armoraciae* and *Ps. chrysocephala* (Figure 5).

## Discussion

The horseradish flea beetle *P. armoraciae* partially prevents the hydrolysis of ingested glucosinolates by plant myrosinases and is able to accumulate large amounts of these defense compounds in the body, mainly in the hemolymph (Sporer et al., 2020; Yang et al., 2020; Sporer et al., 2021). Here, we demonstrate that uptake of glucosinolates from the gut lumen is rapid and selective. We also provide evidence that *P. armoraciae* is able to absorb glucosinolates across the foregut epithelium, a tissue not previously reported to mediate uptake of hydrophilic compounds.

The major functions of the insect foregut are uptake, mechanical grinding, and temporary storage of food before its transfer through the proventriculus to the midgut, where enzymatic digestion and nutrient absorption take place (Chapman, 2013; Denecke et al., 2018; Holtof et al., 2019). Although the permeability of the foregut is generally considered to be very low (Maddrell and Gardiner, 1980), some insect species have been reported to absorb lipophilic compounds, such as cholesterol from the crop (Hoffman and Downer, 1976; Joshi and Agarwal, 1977). In addition, lipophilic insecticides have been shown to be taken up into foregut tissue of the honeybee *Apis mellifera* (Conner et al., 1978). The foregut may even play a role in insecticide detoxification, as treatment of the cockroach *Periplaneta americana* with the neonicotinoid insecticide cycloxaprid resulted in an upregulation of 22 putative xenobiotic detoxification genes in foregut tissue (Zhang et al., 2018). Another study proposed that a sugar receptor that is strongly expressed in the foregut of larvae of the cotton bollworm *Helicoverpa armigera* (Noctuidae) is involved in regulating feeding behavior (Xu et al., 2012). However, compared to the midgut, the foregut is a poorly studied part of the insect digestive system and has been largely excluded from studies of active uptake of hydrophilic compounds.

Adults of *P. armoraciae* were previously shown to absorb a major proportion of ingested glucosinolates within a few minutes from the gut, which demonstrates that glucosinolates are taken up in the anterior part of the digestive tract with an efficient and fast mechanism (Sporer et al., 2021). In this study, we detected ingested glucosinolates in the beetle body when the corresponding plant material was still in the foregut, suggesting that these glucosinolates have been absorbed across the foregut epithelium within less than 1 min after oral uptake (Figure 2). Glucosinolate uptake from the midgut was significantly slower than that from the foregut under our experimental conditions (Figure 2), although we cannot exclude that the injection of a liquid plant extract into the midgut lumen has influenced the glucosinolate uptake rate. Together, our results show that glucosinolates can be absorbed from both the foregut and midgut and suggest that the foregut plays a key role in the uptake of glucosinolates in *P. armoraciae*. Studies with the turnip sawfly *Athalia rosae* also showed a rapid uptake of ingested glucosinolates, which might at least partly take place in the larval foregut (Abdalsamee et al., 2014). However, experimental evidence for this is still lacking. Currently, this phenomenon is insufficiently explored. More species should be investigated to assess the role of the foregut in sequestration of glucosinolates in other groups of insects.

Although both the foregut and the hindgut are lined with a thin cuticular layer, the hindgut intima is much more permeable than that of the foregut as far as known at present (Maddrell and Gardiner, 1980; Chapman, 2013). In the hindgut, water and ions are mainly absorbed from the rectum, which is characterized by a thin epithelium covered with a thin and unsclerotized cuticle (Chapman, 2013). Very little is presently known on the ultrastructure of the foregut of beetles and other insects. Our comparison of the foregut of *P. armoraciae* and two other chrysomelid beetles revealed very distinct differences between the two sequestering and the non-sequestering species, mainly in the structural organization of the chitinous matrix and the overall thickness of the procuticle (Figure 5). These structural differences suggest that the expression level and/or activity of chitin synthases and chitin deacetylases, which influence the amount and structure of chitin in the cuticle (Arakane et al., 2005; Wu et al., 2019; Zhang et al., 2021a), may differ between these leaf beetle species. That the procuticular structure can influence the permeability of insect cuticle was shown in a recent study on migratory locusts (*Locusta migratoria*). Silencing the expression of two chitin deacetylase genes not only increased the susceptibility to infection with an entomopathogenic fungus but also increased the permeability of the cuticle for organophosphorus insecticides compared to a control group (Zhang et al., 2021b). Whether the observed structural differences of the foregut cuticle of sequestering and non-sequestering leaf beetles indeed influence the permeability for hydrophilic compounds like glucosinolates remains to be shown. Comparative studies with a broader sampling may help to reveal mechanisms facilitating the passage and to assess the significance of structural adaptations of the foregut in sequestering and non-sequestering leaf beetles.

*P. armoraciae* absorbed significantly more glucosinolates from the gut than non-host glucosides. This could reflect the substrate specificity of one or several membrane transporters that mediate glucoside absorption from the gut. Several glucosinolate-specific membrane transporters have already been identified in *P. armoraciae*, most of which are localized in the Malpighian tubules. There they prevent the excretion of glucosinolates by selectively reabsorbing them from the lumen of these excretory organs back into the epithelium (Yang et al., 2021). Two glucosinolate-specific transporters with a broad substrate specificity were also expressed in the foregut, but silencing their expression had no influence on glucosinolate uptake (Yang et al., 2021). As our study clearly suggests that glucosinolates are absorbed across the foregut epithelium, we propose that additional transporters in the foregut are responsible for glucosinolate uptake. Gene expression analyses were used to identify more candidates belonging to the MFS transporters, which will be tested in the future (Figure 3; Supplementary Data 1). In addition, transporters belonging to other families could also play a role in glucosinolate absorption in the foregut. We will thus extend the tissue-specific expression analysis to other membrane transporter families in future studies. For example, ATP binding cassette (ABC) transporter family could be involved in the export of glucosinolates from epithelial cells, as this family of membrane transporters is known to be involved in sequestration in other leaf beetle species (Strauss et al., 2013; Schmidt et al., 2019; Kowalski et al., 2020).

Although our study is mainly focused on transport, we additionally made interesting observations regarding the metabolic fate of ingested glucosides in *P. armoraciae* (Figure 1B). For example, from the three tested glucosinolates, we recovered significantly less 2-propenyl glucosinolate in our extracts, which suggests that a higher proportion of this glucosinolate has been metabolized. In addition, we recovered significantly less salicin and catalpol compared to linamarin. The different recovery rates might reflect the substrate preferences of β-thioglucosidase (myrosinase) and β-O-glucosidase enzymes present in *P. armoraciae*. For instance, the previously identified myrosinase from *Phyllotreta striolata* showed β-thioglucosidase as well as β-O-glucosidase activity, although the highest enzyme activity was detected in assays with 2-propenyl glucosinolate as a substrate (Beran et al., 2014). Of course, we cannot rule out that other pathways contribute to glucoside metabolism in *P. armoraciae*.

In summary, our data revealed a rapid and selective glucosinolate uptake mechanism in *P. armoraciae*, which contributes to the selective accumulation of glucosinolates in this insect. We demonstrated an unusual glucosinolate uptake mechanism that involves very efficient absorption from the foregut rather than from the midgut. This rapid uptake is reducing the exposure time of ingested glucosinolates to co-ingested plant myrosinases and insect digestive enzymes, and might thus help *P. armoraciae* to prevent glucosinolate hydrolysis.

## Data Availability Statement

The datasets presented in this study are available at the open access data repository of the Max Planck Society (Edmond) under https://dx.doi.org/10.17617/3.8q.

## Author Contributions

Z-LY and FB designed the experiments and wrote the manuscript. Z-LY, FS, VG, SN, and AR performed the experiments. Z-LY, FS, VG, SN, MR, and RB analyzed the data. All authors contributed to the article and approved the submitted version.

## Funding

This project was funded by the Max Planck Society.

## Conflict of Interest

The authors declare that the research was conducted in the absence of any commercial or financial relationships that could be construed as a potential conflict of interest.

## Publisher’s Note

All claims expressed in this article are solely those of the authors and do not necessarily represent those of their affiliated organizations, or those of the publisher, the editors and the reviewers. Any product that may be evaluated in this article, or claim that may be made by its manufacturer, is not guaranteed or endorsed by the publisher.

## Supplementary Material

Supplementary Data 1 The major facilitator superfamily (MFS) gene expression analyzed by RNA-sequencing.

Supplementary Table 1 Multiple reaction monitoring (MRM) transitions for compounds determined using LC-MS/MS.

Supplementary Table 2 μCT parameters.

Supplementary Table 3 Methods and results of statistical analyses.
