# Supplementary material for: Rapid and Selective Absorption of Plant Defense Compounds From the Gut of a Sequestering Insect
Source: Front Physiol. 2022 Mar 3;13:846732. doi: 10.3389/fphys.2022.846732 (PMC8928188; doi:10.3389/fphys.2022.846732)
Supplement: Supplementary Table 1 — Multiple reaction monitoring (MRM) transitions for compounds determined using LC-MS/MS. [file Data_Sheet_1.PDF]

Supplementary Material

1 Supplementary Data

Supplementary Data 1 | The major facilitator superfamily (MFS) gene expression analyzed by RNA-sequencing.

2 Supplementary Tables

Supplementary Table 1 | Multiple reaction monitoring (MRM) transitions for compounds determined using LC-MS/MS.

Compound	Q1 [m/z]	Q3 [m/z]	CE [eV]
2-Propenyl glucosinolate	358	95.9	-60
4-Methylsulfinylbutyl glucosinolate	435.9	95.8	-60
4-Hydroxybenzyl glucosinolate	424	95.9	-60
Linamarin (formiate adduct)	292	45	-26
Salicin	285	123	-18
Catalpol (formiate adduct)	407	199	-18

Supplementary Table 2 | µCT parameters.

Species	Source voltage (kV)	Source current (µA)	Exposure time (ms)	Image pixel size (µm)	Rotation step (degrees)
Phyllotreta armoraciae	30	170	1600	2.1	0.1
Psylliodes chrysocephala	40	300	1800	1.4	0.15
Phaedon cochleariae	40	300	1800	1.8	0.15

Supplementary Table 3 | Methods and results of statistical analyses.

Comparisons	Transformation	Method	Statistics	*P* value	Figure
Recovery of glucosides	Square-root	One-way ANOVA	F= 22.339	< 0.001	Figure 1B
Sequestration of glucosides	Arcsin-square-root	Generalized least squares	LR = 96.645	< 0.001	Figure 1C
Sequestration of glucosides	–	One-way ANOVA	F= 97.140	< 0.001	Figure 1D
Uptake of glucosinolate	–	Mann-Whitney U test	U = 0.000	< 0.001	Figure 2B
Gene expression	–	Mann-Whitney U test	U = 8922.000	0.136	Figure 3A
